# A High-Q AFM Sensor Using a Balanced Trolling Quartz Tuning Fork in the Liquid

**DOI:** 10.3390/s18051628

**Published:** 2018-05-19

**Authors:** Yingxu Zhang, Yingzi Li, Zihang Song, Rui Lin, Yifu Chen, Jianqiang Qian

**Affiliations:** 1 School of Instrumentation Science and Opto-electronics Engineering, Beihang University, Beijing 100191, China; hopeyxzhang@buaa.edu.cn; 2 Key Laboratory of Micro-nano Measurement-Manipulation and Physics, Beihang University, Beijing 100191, China; zhsong@buaa.edu.cn (Z.S.); linrui@buaa.edu.cn (R.L.); dandingcyf@buaa.edu.cn (Y.C.); qianjq@buaa.edu.cn (J.Q.); 3 School of Physics and Nuclear Energy Engineering, Beihang University, Beijing 100191, China

**Keywords:** atomic force microscope, liquid environment, balanced trolling quartz tuning fork, high quality factor, dynamic mechanical behavior

## Abstract

A quartz tuning fork (QTF) has been widely used as a force sensor of the frequency modulation atomic force microscope due to its ultrahigh stiffness, high quality factor and self-sensing nature. However, due to the bulky structure and exposed surface electrode arrangement, its application is limited, especially in liquid imaging of in situ biological samples, ionic liquids, electrochemical reaction, etc. Although the complication can be resolved by coating insulating materials on the QTF surface and then immersing the whole QTF into the liquid, it would result in a sharp drop of the quality factor, which will reduce the sensitivity of the QTF. To solve the problem, a novel method, called the balanced trolling quartz tuning fork (BT-QTF), is introduced here. In this method, two same probes are glued on both prongs of the QTF separately while only one probe immersed in the liquid. With the method, the hydrodynamic interaction can be reduced, thus the BT-QTF can retain a high quality factor and constant resonance frequency. The stable small vibration of the BT-QTF can be achieved in the liquid. Initially, a theoretical model is presented to analyze the sensing performance of the BT-QTF in the liquid. Then, the sensing performance analysis experiments of the BT-QTF have been performed. At last, the proposed method is applied to atomic force microscope imaging different samples in the liquid, which proves its feasibility.

## 1. Introduction

Since the atomic force microscope (AFM) was introduced in 1986 [1], it has been an important tool in nanotechnology to simultaneously achieve high-resolution topography imaging [2,3] and quantify the physical properties of samples [4,5] in various environments. For example, the AFM can be used to image and manipulate biological samples on atomic even submicroscopic scales in buffer solution [6] and explore the nature of the ionic liquids [7]. In in situ imaging samples in ionic liquids, the force applied to the samples should be as small as possible to protect samples from deformation and destruction, which begets sufficiently precise force control [8]. Therefore, frequency modulation atomic force microscope (FM-AFM) is becoming a better choice to image samples in the liquid because of its super high resolution and force sensitivity [9].

A key for the AFM to achieve ultra-high resolution is the high sensitivity of the sensor, which can be reflected by its quality factor (Q-factor). The Q-factor can be calculated by Q = $m\omega c^{-1}$, where *m* is the mass of the sensor, ω is the angular frequency of the sensor, and *c* is the damping coefficient. Generally, a cantilever is used as the force sensor in a commercial AFM, and the deflection of the cantilever induced by the tip-sample interaction force can be detected by a four-quadrant photoelectric detector. In order to image samples in liquids, the whole cantilever should be immersed into the liquid [10]. However, the Q-factor of the cantilever will be reduced by more than two orders of magnitude, which means a severe decrease in the sensitivity and the measurement accuracy. This is mainly caused by the hydrodynamic interaction between the cantilever and the liquid [11].

After quartz tuning fork (QTF) was first reported in 1989, it has been widely used as the force sensor in scanning probe microscope because of its ultrahigh stiffness, frequency stability, high quality factor and self-sensing nature [12]. When a QTF is used as a force sensor of the AFM, a probe is glued on one of its prongs and the probe-sample interaction results in the changes of QTF’s resonance frequency $f_{0}$ and vibration amplitude *A*. In the vacuum, the force measurement with the QTF can reach femtonewton level [13] and it only attenuates to the piconewton level in the air or the liquid [14]. The advantages of the QTF allow it to achieve high-resolution [15], to obtain biological topography [16] and to analyze the room-temperature ionic liquid [17]. However, QTF’s application is limited by its bulky structure and exposed surface electrode arrangement [12]. In the liquid, the vibration of the QTF will be influenced by the probe–liquid interactions (the hydrodynamic interaction between the QTF and the liquid ) including the Stokes drag force, the meniscus force, and the added mass, which will lead to a significant decline of the Q-factor. The low Q-factor reduces the signal-to-noise ratio of the AFM to make it difficult to image nanostructures of the sample. Moreover, it has been proved that the Q-factor of the QTF with two symmetrical prongs oscillating freely is greater than that of sensors in asymmetrical structures [18]. Therefore, it is essential to decrease the hydrodynamic interaction and improve the symmetry of the QTF by an efficient and convenient method to recover the Q-factor.

**Figure 1:**
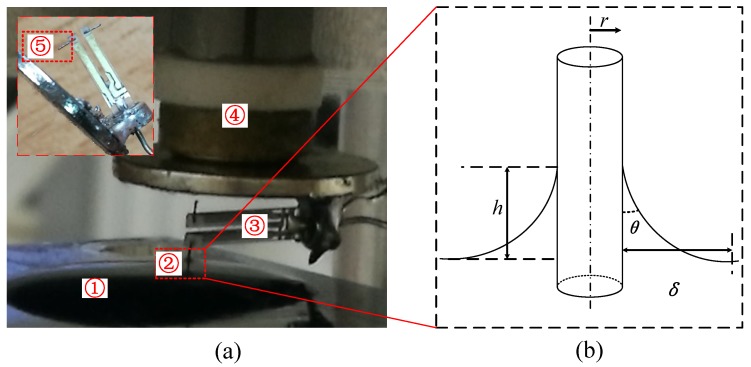
(**a**) Frequency modulation atomic force microscope equipped with balanced trolling quartz tuning fork. ① water, ② tungsten probe, ③ balanced quartz tuning fork with two prongs oscillating freely in the air, ④ piezoelectric tube scanner, ⑤ the balanced tungsten probe. Inset: The close up image of the BT-QTF; (**b**) schematic of the probe in the liquid.

In this paper, a novel method for using the QTF in the liquid, called balanced trolling QTF (BT-QTF, shown in Figure 1), to acquire a high Q-factor is presented. The method is based on a commercially available 32.768 kHz QTF, where the same two probes are glued on both prongs of the QTF separately to improve symmetry. To decrease the hydrodynamic interaction, only one of the probes is immersed in the liquid while the two prongs can vibrate freely in the air. Firstly, the theoretical model is established to analyze the sensing performance of the BT-QTF in the liquid. Then, the sensing performance analysis experiments of the BT-QTF have been conducted, and the influence of the liquid on the Q-factor and resonance frequency of the BT-QTF have been discussed in detail. Finally, the images of actual samples have been obtained in the liquid with the proposed method to prove its feasibility.

## 2. Theoretical Model

A commercial QTF (32.768K, Shenzhen Jinghong Electronics Co., Ltd., Shenzhen, China) with a resonance frequency of 32.768 kHz, whose shell is stripped, is used as a force sensor (Physical parameters of the QTF are shown in Table 1). The tungsten probe is manufactured by the single liquid film etching method developed by ourselves and a probe bonding apparatus is used to help glue the tungsten probe to the QTF [19]. Briefly, the tungsten wire passed through a 1.5 mol/L NaOH solution film (formed in the iron coil) first, and then immersed into a 1 mol/L NaCl solution. The electrochemical corrosion will only occur in the NaOH film and auto-stop after the tungsten wire is broken. Here, the cyanoacrylate is selected as the adhesive due to its ability of instant adhesion. In order to improve symmetry of the QTF, the same two probes are glued on both prongs of the QTF separately. In addition, only one of the BT-QTF’s probes is immersed in the liquid while the remaining part is kept outside of the liquid to eliminate the hydrodynamic interaction between the prongs and the liquid. The study of the dynamics of the BT-QTF in the liquid would help understand the sensing performance and guide the imaging process of the FM-AFM in the liquid.

### 2.1. Probe–Liquid Interactions

When the BT-QTF is used to image, only one of the probes is immersed in the liquid while the remaining part is outside of the liquid. The interaction between the liquid and the probe must be considered in the kinematics equation to learn the influence of the hydrodynamic interaction on the QTF’s vibration. For a daily used tungsten probe with a diameter of 100μm, the non-stationary and convective term in the Navier–Stokes equation can be ignored because the Reynolds number is low enough (Re≪1) [21]. Therefore, the viscous force dominates the fluid motion (Stokes Law), and the Stokes drag force is proportional to the velocity. The motion of the probe in the liquid can be considered as a cylinder moving along its longitudinal direction, and the Stokes drag coefficient can be approximated as [22]:

(1) $$c_{d}=2\pi \eta l{\left[\ln\left(l/r\right)-\left(1.30-8{\left(\frac{1}{\ln(l/r)}-0.30\right)}^{2}\right)\right]}^{-1},$$

where *l* is the length of the probe immersed in the liquid, *r* is the radius of the tungsten probe, and η is the dynamic viscosity of the liquid.

A meniscus, shown in Figure 1, is formed at the probe–liquid–air interface due to the tension of the liquid surface tension, which will exert a capillary force on the probe. To obtain the best signal-to-noise ratio for the FM-AFM, the vibration amplitude of the QTF should be no more than 1nm and the maximum vibration amplitude, where atomic resolution is obtained, is A=380pm in pure water [23] (QTF’s ultrahigh stiffness ensures that it can produce stable small amplitude vibrations in the air and the liquid). Therefore, the mechanical behavior of the meniscus can be seen as a mechanical oscillator with a spring constant $k_{m}$ and a damping coefficient $c_{m}$. The properties of the meniscus have been studied by mechanical behavior of a fiber in the liquid environment, and its spring constant can be expressed as [24]:

(2) $$k_{m}=2\pi \gamma \sin\theta {\left\{\sin\theta \left[\ln(\frac{4\kappa^{-1}}{r(1+\sin\theta )})-0.57\right]+\frac{\cos^{2}\theta }{1+\sin\theta }\right\}}^{-1},$$

where γ is the liquid surface tension, θ is the contact angle between the liquid and the probe, 0.57 is the Euler constant, and $\kappa^{-1}=\sqrt{\gamma /\left(\rho_{\mathrm{liq}}g\right)}$ is the capillary length, which is much larger than the meniscus size in the BT-QTF so that the gravitational effect can be ignored. Furthermore, the damping coefficient of the meniscus is approximated as [25]:

(3) $$c_{m}=2\pi r\eta \ln\left(\frac{\delta }{a}\right)\theta^{-1},$$

where $\delta =\sqrt{2\eta /\rho_{liq}\omega }$ is the evanescent length, ω is the oscillation frequency of the QTF, θ is the contact angle between the probe and the liquid, and *a* is the diameter of the liquid molecule.

### 2.2. Kinematics Equation of the BT-QTF

Each prong of the QTF can be regarded as a harmonic oscillator, and its two prongs are coupled together through the base. In order to obtain the sensing performance of the BT-QTF, a kinematics equation of the QTF is introduced to analyze the impact [26]. Because the same probes are glued on both prongs of the QTF, the mass difference between two prongs can be ignored. However, the mass of the liquid attached on the probe will cause a mass difference Δm that can be calculated by $\Delta m=\rho \pi \phi^{2}\delta$ [24], where ϕ is the diameter of the tungsten. Furthermore, only one of the QTF’s prongs will be affected by the interaction between the probe and the sample, so external force can be expressed as $F_{1}=F_{\mathrm{drive}}$, $F_{2}=F_{\mathrm{drive}}+F_{\mathrm{tp}}$. Then, the equation can be written as:

(4) $$\begin{array}{cc} m{\ddot{x}}_{1}\left(t\right)+\left(k+k_{c}+k_{m}\right)x_{1}\left(t\right)-k_{c}x_{2}\left(t\right)+c_{\mathrm{air}}{\dot{x}}_{1}\left(t\right) & =F_{\mathrm{drive}}\\ \left(m+\Delta m\right){\ddot{x}}_{2}\left(t\right)+\left(k+k_{c}+k_{m}\right)x_{2}\left(t\right)-k_{c}x_{1}\left(t\right)+\left(c_{\mathrm{air}}+c_{d}+c_{m}\right){\dot{x}}_{2}\left(t\right) & =F_{\mathrm{drive}}+F_{\mathrm{tp}}, \end{array}$$

where *m* is the mass of one prong of the QTF, $x_{1}$, $x_{2}$ are the displacements of two prongs, *k* is the spring constant of the QTF, $k_{c}$ is the coupled spring constant between two prongs, and $c_{\mathrm{air}}$ is the air damping coefficient. This model will be used to describe the feature of the BT-QTF in the liquid, and the results will be presented and discussed in the next section.

## 3. Results and Discussion

### 3.1. Influence of the Liquid on the Q-Factor and Resonance Frequency

**Figure 2:**
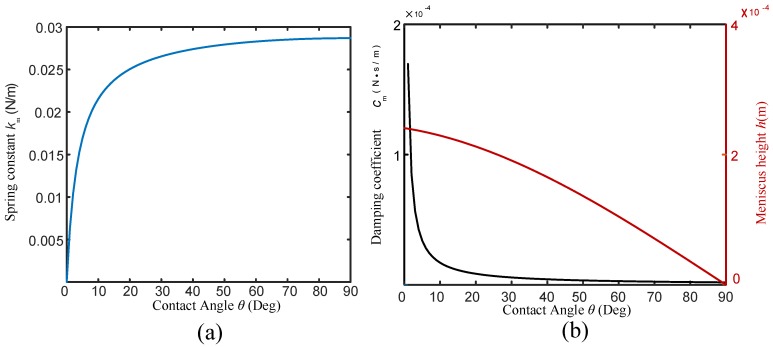
The influence of meniscus on the BT-QTF: (**a**) relation between equivalent spring constant of meniscus on tungsten probe and contact angle, (**b**) relation between the damping coefficient $c_{m}$ or the meniscus height and the contact angle.

**Figure 3:**
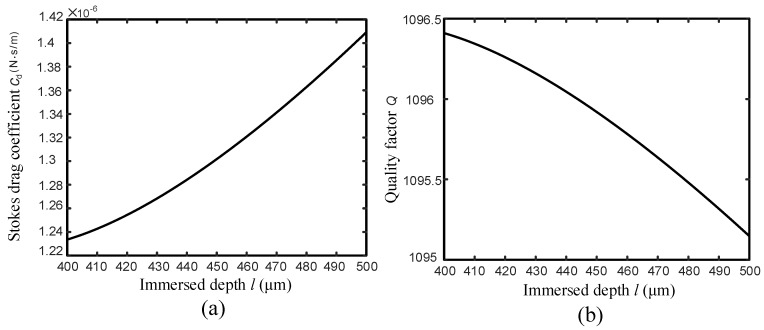
(**a**) relation between the Stokes drag coefficient $c_{d}$ and the probe immersed depth *l* in the liquid, (**b**) relation between the Q-factor and the probe immersed depth *l* in the liquid.

During the FM-AFM imaging, the BT-QTF works at its real-time resonance frequency and scans over the sample. Here, the tungsten wire with a diameter of 100μm is used to help analyze the influence of the liquid on the Q-factor and resonance frequency of the BT-QTF. Because the tip of the tungsten probe is ignorable compared with its dimension, it will be regarded as a cylinder in the following discussion. In order to simplify the discussion, the pure water has been chosen as the experimental liquid and the ’liquid’ refers to the pure water in the following discussion. According to the model presented above, the effect of the meniscus on the probe is shown in Figure 2. In addition, the meniscus is independent of the immersed depth according to Equations (2) and (3). Thus, the effect of the meniscus on the probe can be considered as a constant force acting on the BT-QTF. That is to say, the approaching process of the AFM will not be affected by the meniscus after the probe was partially immersed into the liquid, which is essential to keep the imaging process stable. Generally, the vibration amplitude of the QTF in the FM-AFM is about 100pm [27], which is far less than the capillary length $\kappa^{-1}$ (The capillary length is in millimeters for a tungsten probe with a diameter of 100μm in pure water). Therefore, the meniscus damping coefficient $c_{m}$ will remain constant in the process of imaging. Of course, a large contact angle can efficiently reduce the meniscus damping coefficient. Since the meniscus damping coefficient will be constant after the probe is partially immersed in the liquid, dipping the probe in the liquid before auto-approach can avoid the influence of the meniscus on BT-QTF to simplify the imaging process. The sample topography fluctuates on the nanometer scale so that the relation between the Stokes drag coefficient $c_{d}$ and immersed depth of the probe only needs to be analyzed over a small range, shown as Figure 3a. The Stokes drag coefficient $c_{d}$ only changes 12% even though the dipping length of the probe has been increased by 100μm. It means the change of the $c_{d}$ can be ignored during imaging (In general, the z-direction measurement range of the AFM is on the nano-level), which ensures that BT-QTF can be used for AFM imaging in the liquid.

A Simulink model has been built based on Equation (4) to study the effect of the liquid on the BT-QTF, where the resonance frequency and Q-factor can be calculated by conducting system linear analysis. According to the experimental results, the $c_{m}$ is $3.397\times 10^{-5}\;N\cdot s/m$ and the air damping coefficient $c_{\mathrm{air}}$ is $1.17\times 10^{-4}\;N\cdot s/m$. For a tungsten with a diameter of ϕ=100μm, the $m_{\mathrm{add}}$ is $9.8404\times 10^{-11}\;\mathrm{kg}$. The parameters including $k=2.825\times 10^{4}\;N/m$ and $k_{c}=1.52\times 10^{3}\;N/m$ can be obtained from our previous work [20] and the bonded probe length is set as 1mm. Taking all parameters into the Simulink model, we found that the resonance frequency of the BT-QTF is 29.8420 kHz in the air and 29.8404 kHz immersed in the liquid, where there is a small negative frequency shift. After the probe is immersed into the liquid, the resonance frequency remains constant in regardless of the immersed depth. Then, the relation between the Q-factor and the immersed depth of the probe is presented in Figure 3b where the Q-factor slowly decreases as the immersed depth *l* increases. The relation among three damping coefficients is $c_{d}<c_{m}<c_{\mathrm{air}}$ and the Stokes damping coefficient is far smaller than the air damping coefficient. It can be seen that the BT-QTF minimizes the effect of the hydrodynamic interaction on the QTF. In the approaching and imaging process of the AFM, the Q-factor of the BT-QTF barely changes and the ultrahigh stiffness also prevents the occurrence of the unstable vibrations. The change of immersed depth has no obvious effect on the BT-QTF’s vibration, which guarantees the stability of the instrument and reduces the complexity of control system.

### 3.2. Sensing Performance Analysis Experiments of the BT-QTF

**Figure 4:**
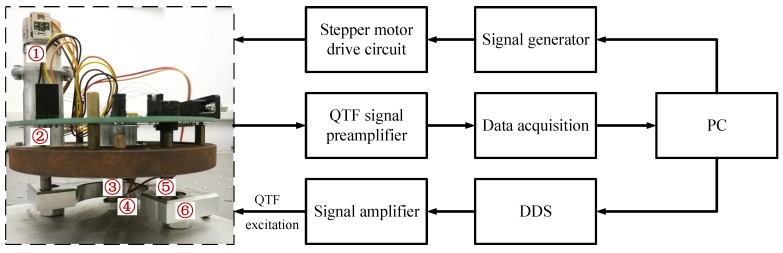
Schematic of the frequency spectrum measurement system for BT-QTF, ① stepper motor, ② transmission device, ③ piezoelectric tube scanner, ④ BT-QTF, ⑤ high-precision screw thread pair, ⑥ support base.

**Figure 5:**
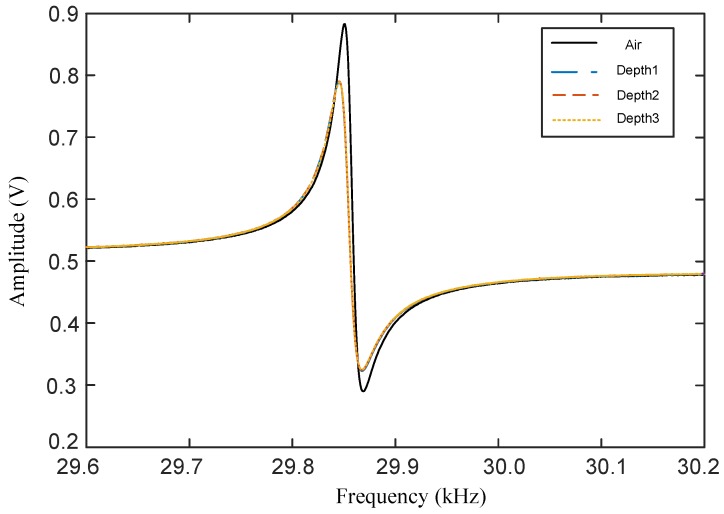
Amplitude-frequency characteristics of the BT-QTF in the air and the probe immersed in pure water with different depths (Δl=10μm). The BT-QTF’s resonance frequency in the air is $f_{\mathrm{air}}=29.851\;\mathrm{kHz}$.

The BT-QTF, where two tungsten probes with length of 1mm are glued on each of its prongs, has been used to conduct experiments. The BT-QTF, fixed at the end of the piezoelectric tube scanner, is assembled in a home-made AFM [28], shown as Figure 4. In order to test the performance of the BT-QTF, a Direct Digital Synthesizer (DDS) is used to drive the BT-QTF. Then, the amplified output of the BT-QTF is acquired by the computer. The displacement of the BT-QTF can be precisely controlled by a stepper motor. The experiment is conducted in the pure water. The dynamics response of the BT-QTF in the air and the liquid with different depths are shown in Figure 5 and Table 2.

It can be seen from Figure 5 that the BT-QTF still maintains a good frequency response in water, although the resonance peak is attenuated. This is because the liquid damping forces including meniscus damping and Stokes damping force will be applied to the probe, which will dampen the movement of the BT-QTF. When the BT-QTF is used in the liquid, the air damping occupies the major position ($c_{d}<c_{m}<c_{\mathrm{air}}$, and $c_{d}\ll c_{\mathrm{air}}$), and the change of Stokes drag coefficient $c_{d}$ with the immersed depth is ignorable compared with the total system damping ($c_{d}+c_{m}+c_{\mathrm{air}}$). Therefore, the frequency response of the BT-QTF almost stays constant after the probe is immersed into water. The Q-factor of the BT-QTF is 1421 in the air and 1065 in the liquid, and there is only a Q-factor reduction of 356, shown as Table 2, which means that the BT-QTF can avoid significant attenuation of the QTF’s sensitivity to meet the requirements of high-resolution and high-sensitivity in the liquid. In different immersed depths, the Q-factor of the BT-QTF remains constant. The resonance frequency $f_{0}$ of the BT-QTF in water would have a −5Hz shift compared with that in air. The deviation of the frequency shift between the experimental value and the theoretical value is mainly because the influence of glue on the BT-QTF is not considered and the added liquid mass cannot be obtained accurately. The resonance frequencies of the probe immersed with different relative depths are the same: $f_{\mathrm{liquid}}=29.846\;\mathrm{kHz}$. The resonance frequency will remain constant in a certain range after the probe is immersed into water, which means that the meniscus is the major factor resulting in frequency shift and Q-factor value decline of the BT-QTF. The influence of the meniscus change on the BT-QTF can be avoided by immersing the probe of the BT-QTF in the liquid all the time, and the change of the Stokes damping coefficient with the depth can be ignored. Thus, the BT-QTF minimizes the hydrodynamic interaction and improves symmetry of the QTF.

### 3.3. Imaging Experiments in the Liquid

**Figure 6:**
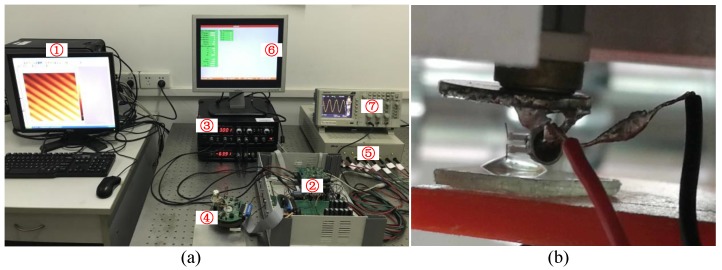
(**a**) the FM-AFM implemented with the BT-QTF. ① Computer, ② The AFM controller, ③ The Nanosurf EasyPLL Plus, ④ The AFM probe, ⑤ The power supply, ⑥ The lower computer monitor, ⑦ The oscilloscope; (**b**) the close-up picture of the BT-QTF during AFM imaging. The thickness of the drop is greater than 1mm to avoid the influence of the liquid evaporation on imaging.

The imaging experiments in the liquid have been carried out to demonstrate the performance of the AFM implemented with the BT-QTF, shown as Figure 6. The vibration signal of the BT-QTF is input to the EasyPLL Plus (Nanosurf AG, Liestal, Switzerland) after it is amplified and denoised. A PC-104 mainboard with a GX1 processor is used to control the scanner, which works under the real-time operating system μC/OS−II and obtains the frequency shift value of the QTF from the EasyPLL plus. Other parameters like setpoint and PID parameters can be set by the operator. Here, a circular microgrid sample and a polymer blend (PS/PB ploymer, Nanosurf), which consists of stiff polystyrene (PS) and soft polybutadiene (PB) on silicon, are imaged by the FM-AFM implemented with the BT-QTF. In order to avoid the influence of the liquid evaporation on imaging (For example, it is more likely that there is at least 500 micrometer liquid over the sample to avoid the concentration of ions due to evaporation while imaging biological samples), a droplet with a thickness greater than 1mm is dropped above the sample. Therefore, a tungsten probe with a length of 3.6mm is glued on the QTF to avoid the contact between the prongs and the liquid. In this condition, the Q-factor and the resonance frequency of the QTF will decline to 898 and 26.928kHz, respectively. Of course, the length of the tungsten probe can be adjusted according to the actual situation, and a shorter tungsten probe is highly recommended under the premise that the measurement requirements have been satisfied.

**Figure 7:**
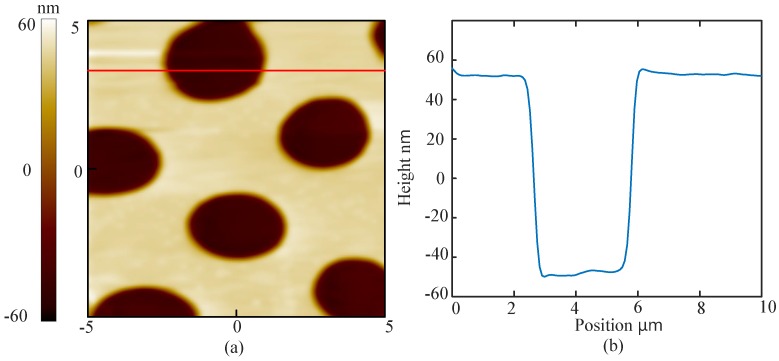
(**a**) topography of the circular microgrid sample imaged by BT-QTF in the liquid; (**b**) the image of the line cross section.

**Figure 8:**
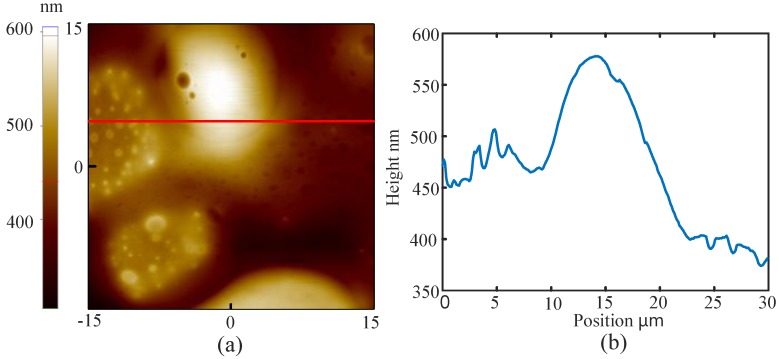
(**a**) topography of PS/PB sample imaged by BT-QTF in the liquid; (**b**) the image of the line cross section.

There are some suggestions of imaging the sample in the liquid using the BT-QTF. At first, an amplitude-frequency characteristic curve of the BT-QTF in the air should be acquired. Then, the probe is controlled to approach the sample and stop after the tungsten probe has been immersed into the liquid. Next, a new amplitude-frequency characteristic curve of the BT-QTF in the liquid must be obtained. To ensure the immersion of the probe, significant resonance frequency shift and resonance amplitude reduction of the BT-QTF should be observed compared with that in the air. Otherwise, the probe should continue to approach the sample until the significant resonance frequency shift and the resonance amplitude reduction occur. It is worth noting that the AFM parameters such as setpoint should be set according to the amplitude-frequency characteristic curve of the BT-QTF in the liquid. Finally, the AFM can be operated as usual. The images obtained by the BT-QTF in the liquid are shown as Figure 7 and Figure 8. It can be seen that the AFM implemented with the BT-QTF can work normally and track the samples well in the liquid. The images of the line cross section (Figure 7b and Figure 8b) show that the BT-QTF can achieve excellent tracking performance at the step and the flat area in the liquid. In addition, Figure 8 shows that the BT-QTF tracks the small PS islands, which are 10–30 nm below the level of the PB matrix and PB islands well in the liquid. It means that the BT-QTF can both track the soft sample surface and the stiff sample surface well in the liquid. In conclusion, the proposed BT-QTF can minimize the influence of the liquid on imaging and work well in the liquid, thus it will reduce the difficulty of the imaging operation in the liquid.

## 4. Conclusions

In this paper, a method called BT-QTF is introduced, through which the hydrodynamic influence can be minimized to maintain high sensitivity of the sensor in the liquid. To analyze its performance, a theoretical model of the balanced trolling quartz tuning fork has been presented. We found that the vibration of the BT-QTF in the liquid is similar to that in the air because the proposed method minimizes the hydrodynamic influence on the QTF and recovers the symmetry of the QTF. The meniscus and the Stokes drag force can be characterized by an equivalent damping coefficient and an equivalent spring coefficient, respectively. The results of simulations and experiments illustrate that there would be a drop in resonance frequency and Q-factor of the QTF, but they will still keep a high value. Since the relation among three damping coefficients is $c_{d}<c_{m}<c_{\mathrm{air}}$ and $c_{d}\ll c_{\mathrm{air}}$, the damping coefficient of the meniscus is the key factor resulting in frequency shift and the decline of the BT-QTF’s Q-factor. Stokes drag force acting on probe can be ignored compared with the total system damping force. The resonance frequency and Q-factor almost do not change with the probe immersed depth in a certain range and QTF’s ultrahigh stiffness prevents the occurrence of the unstable vibrations. Finally, a circular microgrid sample and a polymer blend are imaged by the BT-QTF in the liquid to demonstrate that the proposed method can actually work in the liquid to achieve excellent tracking performance. In addition, some experimental suggestions of using the BT-QTF are given in the paper to help others quickly master it. In addition, the length of the tungsten probe can be adjusted according to the experimental environment.

## Figures and Tables

**Table 1:** Physical parameters of the quartz tuning fork [20]. The geometric dimensions of the QTF were measured by an optical microscopy (SRZ-7045, Beijing Shijikexing Scientific Instruments Co., Ltd., Beijing, China).

Item	Whole QTF	Prong of the QTF
Length (mm)	5.726	3.619
Width (mm)	1.444	0.581
Height (mm)	0.327	0.327
Density ($\;\mathrm{kg}/m^{3}$)	2650	2650
Young’s modulus (GPa)	78.7	78.7
Poisson’s ratio	0.33	0.33

**Table 2:** The experiment results of the probe immersed in different depths.

Depth (μm)	Resonance Frequency (kHz)	Q-Factor
3	29.845	1065
6	29.845	1065
9	29.846	1065
12	29.846	1065
15	29.845	1065
